# Modification of the Dageurreotype Process

**Published:** 1844-12-14

**Authors:** 


### MODIFICATION OF THE DAGEURREOTYPE PROCESS.

M. Fritzsche exhibited to the Academy silver cards, intended to replace the metallic plates at present in use, along with a view procured on one of these cards. M. Lvitsky prepared these cards with Bristol board, which he covers with silver leaf, by means of white of egg and red chalk. They are polished with rouge and cotton. The card is employed in the same way as the metallic plates. Its edges are slightly waxed. The images may be fixed by a concentrated solution of hyposulphate of soda, and the card washed, without injury.—*Polytechnic Review.*

